# Paint It Red - A Multimethod Study of the Nudging Effect of Coloured Cycle Lanes

**DOI:** 10.3389/fpsyg.2021.662679

**Published:** 2021-06-02

**Authors:** Aslak Fyhri, Katrine Karlsen, Hanne B. Sundfør

**Affiliations:** Department of Security, Safety and Behaviour, Institute of Transport Economics, Oslo, Norway

**Keywords:** cycling infrastructure, survey data, GPS-data in bicycle planning, video observation, nudging approach

## Abstract

Many countries colour their cycle lanes, but there is still a lack of research into the impact of this policy. Rather than constraining or regulating movement, coloured asphalt conveys information, and can serve as a good example of a “nudge”. In transport, there are few good examples of effective nudges for improved safety or sustainability. We used a multi-method approach to study the behaviour and experiences of cyclists before and after cycle lanes were coloured using red asphalt. Video data were collected and analysed to measure the extent to which motorists stopped in the cycle lane; motorist distance from the cycle lane on passing; and bicycle placement in the cycle lane. Cyclists (*n* = 1583) were asked how they experienced the cycle lane in field surveys. GPS data from cyclists (*n* = 2448) was used to measure whether colouring the cycle lanes resulted in a change of cyclists’ route choice. Video data showed no significant decrease in the share of passing motorists who stopped in the cycle lane. However, there was a significant decrease in the share of motorists stopping in the cycle lane rather than in the car lane or on the pavement. After recoating, motorists also kept a greater distance from the cycle lane; a greater share of cyclists chose to cycle in the cycle lane and a lower share cycled on the pavement. Analysis of survey data showed that *visibility*, *perceived safety* and *ease of visualisation* improved more in the recoated streets than in control streets. Analysis of the GPS data revealed a significant increase in cycling in the first streets to get red asphalt, with mixed results for the later streets. We discuss possible mechanisms behind the effects observed, and whether coloured cycle lanes can be considered as a form of nudging.

## Introduction

Many countries use cycle lanes, which are created when a line is used to divide road meant for cars from road meant for cyclists. Increasingly, the area meant for cyclists is also distinguished by partial or complete colouring of the asphalt surface. Colouring is intended to make the lanes more visible, thereby increasing other road users’ awareness of cyclists, and making it clear that the area is reserved for cyclists (Høye et al., 2015). Colouring is also thought to increase driver awareness that parking or driving in the lane is not permitted.

Depending on traffic and meteorological conditions, the cost of colouring cycle lanes can be quite high. Different material solutions exist, with the cheapest being to paint the cycle lanes. Problems with paint include the possibility for reduced friction and need for frequent repainting on road stretches with high traffic volumes, or where there is a lot of snow clearance or sweeping of the roadway. Coloured asphalt, or varieties thereof, are more durable and have better friction, but are more expensive. Estimates from Oslo (Norway) indicate a 40% increase in cost, from 25 Euros to 35 Euros per m^2^, for red asphalt compared to black asphalt. From a policy perspective it is therefore important to learn if the increased investment costs pay off in terms of improved safety effects, or other improvements for road users.

### Colour as a Nudge

From a theoretical perspective, coloured cycle lanes can be seen as one of the few good examples of a *nudge* in transport. Nudging was defined by Thaler and Sunstein (2008) and represents a way to influence people’s behaviour without forbidding any options or making them significantly costlier (in terms of money or effort). Nudges work by targeting the shortcuts the human brain takes and should therefore be immediate and often automatic, but cannot be expected to change behaviours people have strong opinions about (Hansen, 2016). Nudging is fundamental in behavioural economics and relies on concepts such as bounded rationality (Simon, 1956) and dual process theory of higher cognition (Evans and Stanovich, 2013) explaining how we are unable to process all relevant information when making decisions. While these systems generally function well, the shortcuts we take are associated with several predictable biases (e.g., framing bias, anchoring effect, default options, automaticity), and changing irrelevant characteristics of a situation can change how we act in those situations (Thaler and Sunstein, 2008).

Due to their ability to maintain people’s freedom of choice, nudges are often easier to implement politically, dealing with changes in the choice context rather than regulations or financial (dis)incentives. Nudges have been found to have effect in the areas of personal savings (Thaler and Sunstein, 2008), health (e.g., Kroese et al., 2015) and household energy-consumption (e.g., Allcott, 2011; Momsen and Stoerk, 2014). In addition, optical illusions are used in road planning to reduce speed at dangerous sections. One example of this is Chicago’s Lake Shore Drive, where lines are painted across the road with progressively smaller gaps between the lines as the curve approaches, giving the illusion that the speed is increasing and thus prompting drivers to slow down.

While many measures are being taken that could qualify as nudges, these measures are usually evaluated without any scientific rigour, or not at all, and there are few publications evaluating nudges in transport (Lehner et al., 2016).

The purpose of colouring cycle lanes is to make the cycle lane more visible and increase the salience of cyclists (Høye et al., 2015), and thus remind both motorists and cyclists that they have designated sections of the road. Just as coloured footprint stickers have been used to guide pedestrians to choose the staircase instead of the elevator (Thaler and Sunstein, 2008), coloured cycle lanes could serve as guides indicating where cyclists are encouraged to be, and so increase the use of cycle lanes and make cyclists behave more predictably.

One particular aspect of coloured cycle lanes that is of interest from a nudging perspective is their potential influence on people’s cognitive maps of the city. Since Kevin Lynch published his seminal work “The image of the city” in 1960 a large body of research has studied how people structure geographical information into cognitive maps. According to Lynch (1960) *routes* constitute important reference points when we orientate ourselves in unfamiliar areas. One study of pedestrians examined the types of information used when giving directions (May et al., 2003) and found that information about distances and street names were the types of information that were *least used* by those who gave directions. Similar studies have not been conducted among cyclists. An interesting research question is whether colouring cycle lanes can help to improve cyclists cognitive map of the cycling network, and in doing so nudge people’s strategic decisions about choice of travel route or mode in everyday life. Colouring cycling network red might make it stand out from the rest of the surroundings, making it more visually salient, and thus mentally salient. This increased salience might then reduce the cognitive load used for navigation and wayfinding, thus allowing cyclists to spend more attention on interacting with other road users.

In addition, the colour used might have a distinct effect, for instance through the colour red’s association with danger, warning or prohibition (Pravossoudovitch et al., 2014). In a recent study, cyclists’ and motorists’ preferences for different colours were examined (Karlsen and Fyhri, 2020). The study showed that green and red cycle lanes were preferred over blue and uncoloured lanes. There was either no significant difference between these, or an interaction where red cycle lanes were preferred by people who were familiar with coloured cycle lanes (which in Norway are red) and green preferred by those who were not familiar with coloured cycle lanes. The study also looked at the potential deterring effect of the warning element inherent in the colour red, but did not find support for this. There was no difference between red and green lanes in people’s expectations about motorists’ violations (stopping or entering the lane).

The most serious cyclist accidents are crashes with cars or trucks (Kaplan et al., 2014), and these crashes are often caused by driver inattention or cyclists behaving unpredictably (Bjørnskau, 2005). If red cycle lanes make cyclists more predictable and motorists more aware, they could be an important contribution to reducing crashes between motor vehicles and cyclists and thus cyclists’ risk of serious injury and death.

### Effects of Coloured Cycle Lanes

Accident analyses of intersections with coloured sections of cycle lanes have been few and inconsistent. While some have found general reductions in accidents (Høye et al., 2015), Jensen (2008) found accident reductions in intersections with one coloured lane, but accident increases in intersections with two or more coloured lanes.

#### Cyclist Opinion and Behaviour

Blue cycle lanes through intersections have been rated as safer than uncoloured lanes by cyclists in both Denmark (Jensen, 2006) and the United States (Hunter et al., 2000). Similarly, red lanes were assessed as safer in Norway (Bjørnskau et al., 2016), though one of the coloured streets evaluated in the latter survey was also wider, which could result in higher safety ratings. As safety concern has been found to be a barrier for cycling (Heesch et al., 2012; Iwińska et al., 2018), especially for women and with increased age (Manaugh et al., 2017), increased feelings of safety might lead to increased cycling in coloured cycle lanes.

A trial from New Zealand examined differences from no cycle lane to an uncoloured lane and then to a coloured lane, finding that while cyclists moved from cycling close to the kerb to 1/3 into the uncoloured cycle lane, the average cyclist cycled in the middle of the cycle lane after it was coloured green (Skilton and Morris, 2013). A study from Alabama did not find a significant difference in cyclists’ distance from the kerb when there was no vehicle present, but did find a significant increase in distance from kerb when cyclists were passed by cars (LaMondia et al., 2019), indicating that cyclists to a lesser degree sought closer to the kerb when being passed in the coloured lane.

Coloured sections in intersections have also been found to have statistically significant effects on cyclist behaviour. In Portland, fewer cyclists slowed or stopped when approaching conflict areas after they were coloured, and fewer cyclists looked behind for motor vehicles (Hunter et al., 2000). In the later evaluation in St. Petersburg, however, there was a doubling in percentage of cyclists scanning for vehicles (Hunter et al., 2008). Two of the evaluations also found significant increases in the proportion of cyclists who followed the coloured sections (Hunter et al., 2000; Brady et al., 2010), meaning that they chose a more predictable position.

#### Motorist Behaviour

Studies looking at coloured lanes in intersections have examined the effect on motorist behaviour using video data. They consistently found an increase in motorists yielding to cyclists (Hunter et al., 2000, 2008), except in one case where many motorists chose to change lanes after the coloured area, though these motorists also increased their use of turn signals (Brady et al., 2010). These studies all used a combination of coloured sections and signs stating “yield to cyclists” (Hunter et al., 2000, 2008; Brady et al., 2010) and therefore cannot isolate the effect of coloured cycle lanes from the effect of the signs.

Cycle lanes in Norway are not coloured in intersections, but rather in full stretches of road. Colouring might be especially important for wide cycle lanes along stretches, as these might more easily be confused with car lanes or parking areas. Statistically significant effects have been found on reductions in vehicle speed and motorists distance to unoccupied cycle lanes (LaMondia et al., 2019), on percentage of motorists driving on the boundary line or in the cycle lane (City of New York Department of Transportation, 2011) and on how much motorists shifted away from the cycle lane when passing cyclists (Skilton and Morris, 2013).

No effects were found for motorists parking in the cycle lane (City of New York Department of Transportation, 2011) or distance between cyclists and passing motorists (Skilton and Morris, 2013; LaMondia et al., 2019), though a trend was found that the smallest passing distances disappeared (LaMondia et al., 2019) and there were non-significant increases in passing distance once the cycle lane was coloured (Skilton and Morris, 2013; LaMondia et al., 2019).

Previous findings seem to indicate that colouring cycle lanes result in motorists increasing their awareness of cyclists and the regulations surrounding cycle lanes, and in cyclists feeling more comfortable on the road. However, the low predictive power of some of these studies does not allow to draw conclusions. As for the reported studies about cyclists, several of these evaluations are local reports rather than peer-reviewed articles, which leaves some uncertainty regarding the scientific quality. Further evidence is needed to evaluate the effects of coloured cycle lanes, particularly regarding motorist behaviour. If car drivers become more aware of cyclists and are reminded of regulations, it should result in them being less likely to park in coloured cycle lanes.

### Research Questions and Hypotheses

The aim of this study was to investigate whether colouring cycle lanes can nudge cyclists and car drivers to change their behaviour, either at the route/mode choice level or at the road user behaviour level. Furthermore, we aim to gain a better understanding of the psychological processes involved when people are exposed to nudge type implementations in real life. More specifically we wanted to investigate if coloured cycle lanes led to

• less cars stopping in the cycle lane
• cars keeping a greater distance from the cycle lane
• improved feelings of safety for the cyclists
• a stronger mental image of the cycling infrastructure
• an increased use of the cycle lane, compared to the other infrastructure
• more cyclists using red coloured lanes than non-coloured lanes.

## Materials and Methods

The present article combines different studies of red cycle lanes into a comprehensive evaluation. We first explain the data collection and procedures for analyses for each, starting with video data, followed by survey data and finally app data.

The app and survey studies were approved by the Norwegian Centre for Research data, while no approval was needed for the video collection due to the low resolution of the images.

### Study Context

The general approach for this project was to work in close co-operation with the city council of Oslo, which during this period had a program for recoating its major cycle infrastructure (cycle lanes) with red asphalt, alongside other improvements and expansions to the network. One result of this cooperation was that we were able to identify the case streets for our study. It should be noted that quite a number of the planned projects had not been completed (in most cases not even started) by the end of the project, so that, in addition to the cases reported here, there were a number of streets where data were only collected for the before situation.

The city of Oslo has for many years had expansive plans for improving its cycling infrastructure to increase cycling levels. These plans, and their fulfilment, were given a boost in 2015 when the Green Party came into office as part of the City Government of Oslo. Counting data from official counting loops (23 on roads governed by the City Council and four on roads governed by the National Public Roads Administration) showed a 4% increase in cycling from 2016 to 2017 and an 11% increase from 2017 to 2018. Still, with a cycling share of 7% (varying from 2% in winter to above 20% in summer), it will be challenging for the city to reach its own and national goals of a 20% cycling share by 2023 (Lunke and Grue, 2018).

### Video Data

**FIGURE 1:**
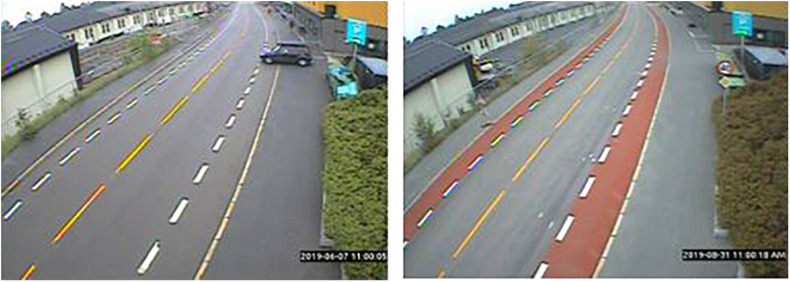
Camera capture at Kongsveien before and after the cycle lane was recoated with red asphalt.

Video data was collected using a Miovision Scout camera. The camera was mounted on a 6-metre-high telescopic pole, that was secured to a lamp-post near the cycle lane to get a bird’s eye perspective of the lane (Figure 1). Effort was made to obtain the same camera placement and angle in the before and after situation. The camera captured footage with a low enough resolution (720 × 480 pixels) to ensure privacy (faces and number plates cannot be recognised).

Due to camera availability, the before period (early June 2019) captured Thursday through Sunday, while the after period (late August 2019) captured Friday through Monday. In general, the camera recorded from 06:00 – 19:00 each day, with some variation across days. More hours were filmed in the before than after period, specifically from 06 to 09 AM. Only matched hours were included in the analyses, giving a total of 82 h of study footage (41 h in each before and after period).

Kongsveien is an arterial road situated in a residential neighbourhood. It functions as an important link for cyclists travelling to the city centre. The segment used as the test area has a speed limit of 40 km/h, and runs in front of a building housing several functions, including a pharmacy, an ATM and bank, and a hair-dresser. The segment was selected because of complaints that cars often stopped or parked in the cycle lane, thus forcing cyclists either onto the pavement or into the car lane. The speed limit and give-way sign in the after picture are not new, but had been moved from just behind the camera to just in front of the camera.

#### Procedure for Analyses

Cars stopping in the cycle lanes were counted before and after the cycle lane was recoated. The street had a few legal parking spots by the side of the buildings and a parking garage underneath; cars stopping in these places were not counted. All cars that stopped on the pavement, in the cycle lane or in the car lane were counted unless their only option was to stop where they did (e.g., delivery trucks too big for the legal parking spots or vehicles associated with road maintenance; vehicles that were forced to stop to make way for emergency vehicles, and so on).

In 5-min intervals each hour (e.g., 09:30–09:35) the distance from cycle lane was recorded for each passing car driving away from the camera (toward the city centre). Cars driving toward the camera blocked the view of the cycle lane they were passing. Distance was measured by counting distance in millimetres on the screen and converting to actual distance. Distance was measured from the back tyre nearest the camera to the border of the cycle lane. We also noted whether cars had just passed or were about to pass cyclists at the point at which their distance to the cycle lane was measured.

To account for exposure, the total number of passing vehicles was also counted. Data on traffic volumes were captured with RUBA software (Agerholm et al., 2017). A presence detector created in RUBA provided a print screen every time an object crossed the detector. These print screens were visually checked to control for false detections.

In addition, cyclists and their placement (pavement, cycle lane or car lane) were manually counted (RUBA software does not reliably count these) for 10 min every hour (e.g., 09:30–09:40). A total of 263 cyclists were counted in the before period and 296 in the after period. It should be noted that cycling on the pavement is permitted in Norway.

### Road Side Surveys

Data were collected in a series of field surveys of cyclists in the intervention streets. The first round of interviews was carried out in 2017 and the second round in 2018. Surveys were carried out in five test streets before and after the intervention (red asphalt implemented). Parallel to this, surveys were conducted in a control street in which no changes were made to the infrastructure. The data collection period spanned over 2 to 3 days at each time point. Interviews were conducted on weekdays, during daytime in the main cycling season (April to October). Most interviews were conducted in the morning and afternoon, during rush hours, in order to recruit enough respondents at each location.

The interviewers were instructed to stop any cyclist approaching them. Each interview took approximately 4–5 min to complete, and data were registered using tablet PCs. Interviews were only conducted on days with no rain. Cyclists were quite eager to respond. No systematic record was made of refusal rates, but an based on using a similar approach in another survey, the refusal rate is estimated to be around 30% (Flügel et al., 2020). This figure is a combination of people not stopping their bike when approached and people having stopped and then declining after having been presented with the survey. Verbal accounts from the interviewers indicate that the most common reason for declining was lack of time. Table 1 shows the number of survey participants at test and control locations.

**TABLE 1:** Overview of survey respondents.

	Before	After
Control	208	245
Test	582	546

#### Survey Items

Only data from streets where red asphalt had been implemented, as well as the control street, were used for analysis. Data corresponding to the different streets or time points were identified by constructing the variables “Condition” (test; control) and “Time” (before; after).

Table 2 shows the variables that were used for analysis.

**TABLE 2:** Survey items included in analysis.

Nr	Name	Item	Scale
1	EaseVisualisation	When I ride this street I can easily visualise my route from one place to another	1; totally disagree, 7; totally agree
2	MentalCost	When I ride this street I have to use a lot of mental capacity to orient myself	1; totally disagree, 7; totally agree
3	EasyDescribe	Imagine someone asked you what route they should follow to cycle from [A to B] and you were to propose this street as part of the route, how easy would it be for you to describe where the person should cycle?	1; very difficult, 7; very easy
4	LikelyRecommend	Imagine someone asked you what route they should follow to cycle from [A to B] how likely are you to recommend just the street we are in now?	1; very unlikely, 7; very likely
5	FeelSafe	I will now ask you about safety. By safety, I mean avoiding traffic accidents. On a scale of 1 to 5, where 1 is very unsafe and 5 is very safe, how safe do you feel it is to cycle in this street (the stretch you just cycled)?	1; very unsafe, 5; very safe
6	KnowPlace	When I cycle in this street I know well where my place is	1; totally disagree, 7; totally agree
7	Visibility	What do you think about the visibility of the bike lane here?	1; very poor; 5; very good
8	Automaticity 1	Choosing this street when cycling in this part of town is my natural first choice	1; totally disagree, 7; totally agree
9	Automaticity 2	Choosing this street when cycling in this part of town is something I do automatically	1; totally disagree, 7; totally agree
10	Automaticity 3	Choosing this street when cycling in this part of the city is something I do without thinking	1; totally disagree, 7; totally agree
11	Automaticity 4	Choosing this street when cycling in this part of town is something I’ve done for a long time	1; totally disagree, 7; totally agree
12	Noticed^1^	To what extent did you notice that you rode on red asphalt on this stretch before we stopped you?	1; to a very little extent, 7; to a very large extent
13	Important	How important was it that it was red asphalt on this lane for your choice to cycle this route?	1; very unimportant, 7; very important

*^1^Only descriptive statistics are presented for these as they were only asked in the test streets after they were coloured red.*

Items 1 to 3 were intended to capture the degree to which the red asphalt gave a more salient image in people’s mental maps of the city. Items 4 to 7 were based on previous surveys, and captured feelings of safety and interplay with other road users, as well as more general impressions of the street. Items 8 to 11 (only asked in the before and after surveys in 2018) are based on the Self-Report Habit Index (SRHI) (Verplanken and Orbell, 2003), and intend to capture the underlying factor “default option/automaticity”. Initial testing showed that items 8 to 11 have a high reliability (Cronbach’s α = 0.81), so the mean score of these four items was used. The final two items were asked only after lanes were coloured, and capture the degree to which the cyclists had noticed the red asphalt and whether it was important for them^2^.

In addition, background questions about amount of cycling and how often people cycled in the given street were asked. The interviewers registered gender and age of the respondents. Table 3 shows important background characteristics for respondents before and after in control and test streets.

**TABLE 3:** Background characteristics for respondents before and after in control and test streets per cent.

	Control before	Control after	Test before	Test after
Age mean (SD)	45 (13)	46 (12)	40 (11)	42 (12)
Gender (women)	46.2	52.4	45.0	40.6
Cycle in street				
>three times per week	78.8	76.4	77.3	80.3
<three times per month	11.5	11.8	10.8	11.5

#### Procedure for Analysis

Survey items were analysed using two-way between group ANCOVAs, where age and gender were included as covariates. Respondents with missing age responses (*N* = 13) were excluded from the analyses. Analyses were conducted using IBM SPSS v. 26 with an alpha level of 0.05.

### App Study

To record changes in amount of cycling we used data collected with a mobile phone app Sense. DAT from Mobidot. This is “self-learning” travel behaviour app that maps route choices and mode choice for travel outside the house. The app uses the phone’s positioning service to locate the mobile, by cellular network, Wi-Fi network, GPS data, or a combination of these. The measured positions are projected into an OpenStreetMap network. The automatic categorisation of travel modes is based on an algorithm that looks at the characteristics of the individual trip, such as speed and route selection. In addition, it can utilise several other sensors in the mobile phone, such as accelerometers. The algorithm has a reported accuracy of 90% when identifying bike trips (Thomas et al., 2018).

As a basis for the analyses in this study, we have chosen to use data from three data collections (*n* = 2448) with Sense.DAT, in 2016, 2017, and 2018. In addition to the app data, we use counting data from relevant counting loops to calibrate the measured changes in amount of cycling toward general trends in cycling. Details about these data have previously been reported (Fyhri et al., 2019), so only a brief summary is given here.

The first dataset comes from a study of cyclists and potential cyclists in 2016. The data collection with Sense. DAT started on April 1 and ended on June 30. There were a total of 728 users of the app.

The second set of data came from a national collaborative project between TØI and a number of Norwegian bicycle towns, in order to increase the understanding of bicycle use in the cities. The project took place in September 2017, and data from 873 app users in Oslo that were used in the analyses.

The third dataset came from the two Norwegian Research Council projects “Cycle to Zero” and “Push and Show”. A total of 845 had used the app Sense. DAT in Oslo.

#### Procedure for Analysis

Based on the communication with Oslo city council, we identified all road sections that had replaced black asphalt with red asphalt in the period between the app surveys. Projects that were in progress during the survey periods were omitted.

The relevant road sections were drawn as polygons in a separate map layer in QGIS. Bike rides were drawn out as lines and the number of lines and the number of kilometres of lines that intersected a road were then counted. To reduce random errors from poor GPS signals etc, and to remove cyclists who simply crossed the test streets, we set 50 metres as the lower threshold of how much of a GPS trace should intersect with the test street to be included as a cycling activity in that street. Sensitivity analysis of five of the case streets showed that this filtering reduced the number of passing cyclists by between 30 and 60%, and the number of cycled kms by between 3 and 9%, indicating that most of the removed traces were from crossing cyclists or from random errors.

The app data collected was used to measure whether there was increased cycling in coloured cycle lanes. We present the results of the analyses for each year separately. In total we have data from 2448 app users, but the number of users in the sample varies somewhat from year to year. This is partly due to the recruitment methodology and partly due to the different duration of data collection periods. Other factors such as weather, time of year and general changes in cycling levels can also influence the results. To account for this, we standardised the changes from the first year to the second, so that they are seen in relation to the total number of cyclists in the samples. The normalised change in cycling was calculated using the expression below, where T is cycling level (either kms or passes) in the test street and O is total cycling in Oslo (thus functioning as a control area), and _1_ indicates “before” and _2_ “after”.

(T_2_/O_2_/)(T_1_/O_1_)

## Results

The results are presented according to the method of data collection, starting with results from the video data, followed by survey data and concluding with the results of the app data.

### Video Data

#### Cars Stopping and Parking

When the cycle lane was uncoloured, 223 out of 11 352 passing cars stopped in the cycle lane. After it was recoated with red asphalt, 213 out of 11 946 cars stopped in the cycle lane. This reduction from 2.0% to 1.8% was not significant and there was not a consistent pattern across days regarding percentage of cars stopping.

**FIGURE 2:**
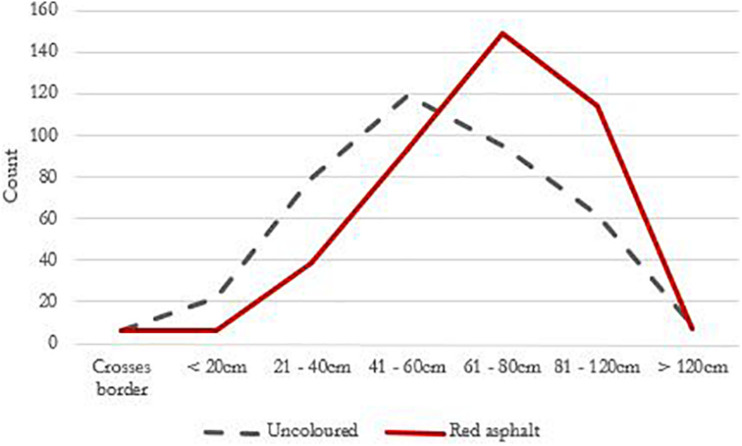
Distribution of motorists’ distance to cycle lane border before and after.

While there wasn’t a significant reduction in the share of cars that stopped in the cycle lane, we also noted cars that stopped outside the cycle lane. Figure 2 shows the percentage of cars that stopped in- versus outside the cycle lane in the two conditions.

A chi-square test of independence was performed to examine the relationship between where cars stopped and the colour of the cycle lane. Motorists were more likely to park outside the cycle lane when it was red than when it was uncoloured and this difference (6.9% points) was significant [*X*^2^(1, *N* = 497) = 5.502, *p* = 0.019]. This was a consistent pattern across all 4 days.

#### Car Distance to the Cycle Lane

**FIGURE 3:**
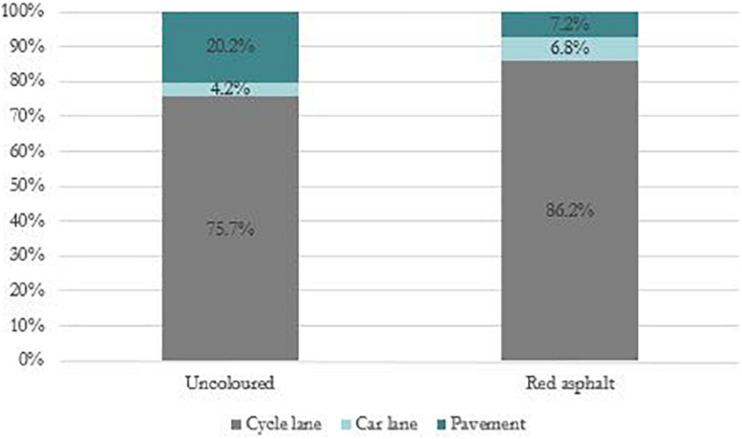
Percentage of cars stopping in- versus outside of the cycle lane before (*n* = 244) and after (*n* = 252) the implementation of red asphalt.

The distance was measured on 392 cars passing the cycle lane before it was recoated, and 414 cars passing after. Figure 3 shows the distribution of distances before and after, revealing that there was a reduction in the shortest passing distances as the whole curve moved further away from the cycle lane.

The mean distance (cm) was greater after the cycle lane was recoated with red asphalt (*M* = 68.34, *SD* = 24.52) than when it was uncoloured (*M* = 58.95, *SD* = 28.13). This difference (9.40 cm) was significant [*t*(775,73) = 5.062, *p* = 0.001].

We also noted whether cars had just passed or were about to pass cyclists when their distance to the cycle lane was measured. There were only 13 cases before and 11 cases after the cycle lane was recoated. Though there was a difference in mean distance (cm) from before (*M* = 107.69, *SD* = 34.46) to after (*M* = 114.55, *SD* = 36.98), this was not statistically tested due to the small sample size.

#### Cyclist Placement

**FIGURE 4:**
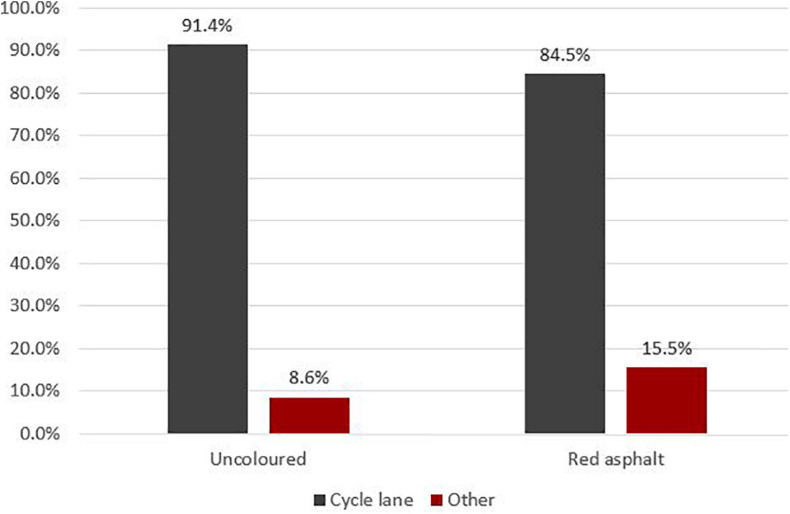
Distribution of cyclist placement before and after recoating with red asphalt, *N* = 559.

We wanted to examine whether there would be a higher share of cyclists using the cycle lane after it was recoated with red asphalt. Figure 4 shows the distribution of where cyclists cycled in both conditions.

Even though most cyclists (75.7%) used the cycle lane before it was coloured, there was a significant (*p* = 0.002) increase (to 86.2%) after it was recoated with red asphalt. There was also an increase in cyclists using the car lane, from 4.2% to 6.8%, but this difference was not significant (*p* = 0.18). There was a clear decrease in cyclists using the pavement, from 20.2% to 7.2%, and this difference was statistically significant (*p* < 0.001). This was a consistent pattern across all 4 days.

### Roadside Survey

To examine the effect of colouring the asphalt on cyclists’ perceptions of the cycle lane, we analysed the responses to several field surveys.

The first items analysed covered cyclists’ perceptions about cycling in the street, more specifically their feeling of safety, knowing one’s place in the street, degree of visibility of the cycle lane, and how likely they were to recommend the street to others. Table 4 shows mean scores before and after for test and control streets, the relative difference between change for control and test streets, and F-test values for the interaction term for time x condition with associated significance levels.

**TABLE 4:** Perceptions about cycling in the street, before and after for test and control streets.

		Visibility	Feel Safe	Know Place	Likely Recommend
Control	Before	3.27	3.72	6.00	6.00
	After	3.40	3.72	6.10	5.98
Test	Before	3.46	3.77	6.10	5.99
	After	4.48	4.00	6.36	6.20
	Relative difference	0.90	0.23	0.15	0.23
Interaction time* condition	*F*	59.87	4.38	0.70	2.24
	Sig	<0.001	0.037	0.403	0.135
	*N*	1574	1574	1574	1574

*Mean scores and relative difference between change for control and test streets. *F* test values for the interaction term for time and condition with significance levels and *N*.*

The items LikelyRecommend and KnowPlace are quite skewed as most people agreed with these statements in both the control street and the test streets. For the other two items the scores were more normally distributed. Cyclists in streets with red cycle lanes feel safer than cyclists in non-coloured lanes, and perceive the cycle lane to be more visible. The difference in increased feeling of safety between the test streets (0.23) and the control streets (0.00) was statistically significant: *F*(1,1568) = 4.38, *p* = 0.037. The difference in increased ratings of visibility between the test streets (1.02) and the control streets (0.13) was statistically significant: *F*(1,1568) = 59.87, *p* < 0.001. There were no significant differences in knowing one’s place in the street or recommending it to others.

The second group of items investigated the street as a default option and the streets’ salience in cyclists’ mental image of the city. These questions (except how easy it is to describe the route) were only asked in 2018. Table 5 shows mean scores before and after for test and control streets, as well as relative difference between change for control and test streets, and F test values for the interaction term for time and condition with associated significance levels.

**TABLE 5:** Perceptions about cycling in the street, before and after for test and control streets.

		Automaticity	Easy visualisation^1^	Easy describe	Mental cost^1^
Control	Before	5.88	1.92	5.38	4.24
	After	6.00	1.98	5.70	4.65
Test	Before	5.84	3.86	5.71	3.33
	After	6.04	4.63	5.97	3.28
	Relative difference	0.08	0.71	−0.05	−0.46
Interaction time*condition	*F*	0.16	4.08	0.06	2.59
	Sig	0.688	0.044	0.811	0.108
	*N*	664	1238	1574	1238

*Mean scores and relative difference between change for control and test streets. *F* test values for the interaction term for time and condition with significance levels and *N*. ^1^Slight change in wording between surveys.*

The items Automaticity and EasyDescribe were quite skewed as most people agreed with these statements. For the other two items the scores were more normally distributed. Cyclists in streets with red cycle lanes find it more easy to visualise the street than cyclists in non-coloured lanes. The difference in increased ease of visualisation between the test streets (0.77) and the control streets (0.06) was statistically significant: *F*(1,1232) = 4.08, *p* = 0.044. The mental cost and automaticity variables changed in the hypothesised directions but the changes were not statistically significant. There was an increase in ease of describing where to cycle for both test and control streets and there was no interaction between time and condition for this item.

Respondents who were stopped on a red cycle lane (*N* = 546) were asked to indicate on a scale from 1 to 7 the extent to which they had noticed that the lane was red and how important the red asphalt was for their choice of using that street. Most of the participants had noticed that the cycle lane was coloured red and 66.4% gave the highest score on this question (*M* = 5.75). There were, however, some (10.8%) who gave the lowest score (1). When asked about the importance of red asphalt, quite a lot (37.1%) gave the lowest score, but there were also some who said it was “very important” and gave it the highest score (13.9%); the mean score for this item was 3.3.

### App Study

As mentioned, we calculated the normalised change in cycling, thus accounting for the general trend for cycling levels in Oslo in the selected study periods.

To illustrate the general extent of cycling in the street in question, the tables also present the number of kilometres cycled and the number of cycle passes in the individual streets in the first year. The standardisation means that the ratio of the individual street to the total number of cyclists is set to 1.00 in the before situation. Numbers above 1.00 indicate a relative increase in the number of cyclists, whereas numbers below 1.00 indicate a decrease.

Table 6 summarises changes in the number of kilometres cycled and the number of passing cyclists in groups of streets that have implemented red asphalt, as a function of the total number of cyclists in Oslo. The table shows absolute numbers in cyclists in the before situation (2016 or 2017) and index values for change in the after situation (2017 or 2018).

**TABLE 6:** Changes in the number of kilometres cycled and the number of passing cyclists in streets that have received red asphalt, as a function of the total number of cyclists in Oslo.

Year	Measure	Number of case streets	Kilometre cycled before	Passes before	Kilometre, change after	Passes, change after
2016–2017	Red asphalt	3	845	2289	1.19	1.13
2016–2017	Red asphalt + wider lane	5	2086	3674	1.38	1.27
2017–2018	Red asphalt	4	921	2468	0.93	0.92
2017–2018	Red asphalt + wider lane	1	253	673	1.37	1.04
	Total	12	4105	9104	1.12	1.03

*Absolute numbers in in the before situation (2016 or 2017) and index values in the after situation (2017 or 2018).*

Overall for the two periods, the streets that were coloured with red asphalt had a 12% (index value 1.12) increase in kilometres cycled compared to the rest of Oslo.

From 2016 to 2017 the three streets with only red asphalt had a relative increase of 19% in kilometres and 13% increase in cyclists passing compared to the rest of Oslo. The change for the five streets with red asphalt and width increase was larger, with 38% in kilometres and 27% increase in cyclists passing. Of the eight streets included, all but one experienced an increase in cycling. The street with a decrease was close to a connecting street undergoing an upgrade in the period, which might explain this result.

In the next round of implementing red asphalt (2017–2018), results were a bit more mixed. The four streets with only red asphalt had a relative decrease of 7% in kilometres and 8% (index values 0.97 and 0.92) in cyclists passing. Of the four streets, two experienced an increase and two had a decline in kilometres cycled and cyclists passing. The one street with red asphalt and increased width had an increase of 37% in kilometres and 4% in cyclists passing.

## Discussion

The present study examined whether red cycle lanes relate to changes in behaviour for motorists and cyclists, as well as changes in cyclists’ opinions about the cycle lane. Using different methods and different streets in different years, the generally cohesive results give a strong impression of the benefits of red cycle lanes.

Video observation analyses indicate a small reduction in percentage of cars stopping in the cycle lane that was not statistically significant. The video camera recorded 4 days and there was quite a bit of variety between the different days. Future research should consider that both day of the week and traffic amount can influence the rate of cars stopping in the cycle lane, and should ideally capture longer periods of time.

However, there was a significant change in *where* cars stopped. After the intervention, stopping motorists avoided the cycle lane to a greater degree than before, choosing other – also illegal – places to stop. This indicates that colouring a cycle lane works to keep more motorists out of the lane, but is not a strong enough incentive to keep them from stopping at all. As the intervention (colouring) does not include any legal changes it makes sense that people who feel the need to park there (e.g., to drop someone off at the apothecary or get money at the ATM) won’t ignore that need, but rather choose to stop somewhere else, such as in the road next to the cycle lane or on the quite broad pavement. Motorists stopping in the cycle lane represent a safety risk as cyclists are forced to go around, often into the car lane, and will suddenly enter areas where other road users are not expecting them (Bjørnskau et al., 2016) and reducing motorists’ tendency to park in the cycle lane might thus reduce the risk of motor vehicle – cyclist crashes. While motorists avoiding the cycle lane might benefit cyclists, other issues might be created by the fact that motorists instead either stop in the car lane, forcing other cars to enter the opposite lane to pass them, or drive onto the pavement.

As in previous studies (Hunter et al., 2000; Bjørnskau et al., 2016), we found an increase in safety ratings after the cycle lane was coloured, alongside an increase in perceived visibility of the cycle lane. This could be a direct effect of the red asphalt, with cyclists feeling safer in a more visible cycle lane, but also an indirect effect of real or perceived changes in motorist behaviour. While uncoloured cycle lanes themselves are associated with increased cycling (Parker et al., 2011; Ellis et al., 2016) this indicates that colouring the lanes could boost the increased feeling of safety and thus lead to more cycling. However, the results regarding increased cycling in red cycle lanes were inconsistent. While the app data results from 2016 to 2017 showed a clear increase, the results from 2017 to 2018 were mixed, with a decrease in cycling in some streets and increase in others. One explanation for this reduced effect of the later projects is a “novelty” effect where the first streets that were given red asphalt stood out more from the general cycling network and therefore were more mentally salient for potential cyclists. Another explanation is that the streets that were coloured red first were more crucial as “missing links” in the network than the later streets. The city council had a strategy to “pick the lowest hanging fruit first”, i.e., to first roll out the projects where change was most likely to have the most impact on road users.

Another point to consider is that when asked how important the colour was to cyclists’ choice to cycle in the street they were in, more than a third gave the lowest importance, and the mean was 3.1 out of 7. This illustrates that while colouring a cycle lane can have important implications for cyclists’ perception of a street, there are many other factors that combined or separately are more essential for route choice. For instance, the measures of automaticity were quite high and previous research has found habit strength was a stronger predictor of travel mode choice than both intentions and perceived behavioural control (Verplanken et al., 1998).

### Red Cycle Lanes as a Nudge?

As well as evaluating the effect of red cycle lanes, the current study aims to investigate if this can be considered as one of the few proven examples of nudging in transport. Intriguingly, nudges are defined just as much by *whether* they work as how they work (Thaler and Sunstein, 2008; Hansen, 2016). This somewhat tautological definition points to an important aspect of much of the literature concerning nudges; a number of clever interventions sail under the nudging flag, but are yet to be proved with validated methods. The question then is, have we been able to prove that coloured cycle lanes are nudges?

Changing the colour of a cycle lane does not change its legal position or any consequences related to cars or cyclists being in the cycle lane (Høye et al., 2015). However, our results taken together with previous results (e.g., Hunter et al., 2000; LaMondia et al., 2019) indicate that changing the colour of the lane changes both motorist behaviour (stopping in lane and passing distance) and cyclist behaviour (not cycling on pavement, route choice in the first round). Thus, it can be claimed that colouring a cycle lane can be classified as a nudge.

Cyclists and motorist behaviour is complex and interconnected, and these results do not reveal which changes are direct results of the nudge and which are more indirect. As cyclists and motorists interact in traffic, it seems plausible that changes in one can lead to or reinforce changes in the other, resulting in a positive feedback loop. If increased salience leads to motorists keeping out of the cycle lane and cyclists feel safer, this could lead to more cyclists, which again could lead to a “safety-in-numbers” effect (Fyhri et al., 2017; Elvik and Goel, 2019).

We found increases in cycling volumes in the coloured lanes with the app data, albeit with some variations. These data cannot tell if the observed increases (from 2016 to 2017) are from cyclists changing from non-coloured to coloured lanes (route choice) or from people changing from other transport modes to cycling (mode change). The choice of which route to cycle, and choosing whether or not to cycle, are decisions relying on a range of practical, emotional and social considerations. Comparing the two, it is more likely that a simple colour nudge influences route choice rather than mode choice, either directly as a clue about the presence of adequate cycling infrastructure or indirectly via improved (perceived) safety. This interpretation is also in line with a previous study, using the same type of data in Oslo, (Pritchard et al., 2019), that found route changes but no mode share effect from improved cycling infrastructure.

It is more likely that colouring cycle lanes directly influences the distance motorists keep from the lane, as well as their tendency to park in it. The fact that the empirical data regarding perceptions and motorist behaviour cover rather a short time span, supports the presumption that these changes are derived directly from the nudging effect, and not from more secondary indirect effects. While some motorists who stop in cycle lanes may have clear opinions about it, most are probably looking for the most convenient place to reach their goal (e.g., a quick stop for an errand, picking up or dropping off someone). If they’re stopping in the cycle lane because it seems convenient, they might not consider the impact it can have on cyclists. Colouring the cycle lane might increase the salience of cyclists, as well as the law forbidding them to park there. In addition, there might be social norms involved in that a car stopped in a coloured cycle lane is more conspicuous than a car stopped in an uncoloured cycle lane – though this factor was not examined in these studies.

Nudges are defined as changes in a choice context that influence behaviour in predictable ways, and that do so while only changing aspects that are *presumably irrelevant*, i.e., of no consequence for the individual’s rational choice. In this study we have measured the *behavioural outcomes* of the nudges in various ways (motorists passing distance, cyclists placement, change of routes etc.). What is novel, compared to previous studies is that we have also attempted to measure the *intermediary processes* from the nudge to the behaviour, i.e., the mechanisms by which the nudge works. We have attempted to operationalise these processes in the survey by measuring changes in people’s cognitive maps and automaticity of choice (habits) of the affected streets. With the exception of ease of visualisation, none of these aspects changed after the lanes were coloured red. There were some methodological challenges with these items (see below), so it is hard to know if there were any real changes in the automatic choice of using these streets or their salience in people’s mental maps (beyond the observed ease of visualisation). To our knowledge, this study is the first attempt at actually testing the psychological correlates of a nudging intervention. Even if some of the items, such as the SRHI, are previously validated, future research should investigate their *construct validity* related to nudging.

### Strengths and Limitations

To our knowledge, this is the first comprehensive article looking at the effects of coloured cycle lanes. We combined different methods to examine several behaviours and the results generally correspond to each other and paint quite a cohesive picture. In addition, the studies include several different streets and cover several years (2016–2019), strengthening our findings.

While some have previously looked at cyclists’ position relative to the kerb (Skilton and Morris, 2013; LaMondia et al., 2019), we examined whether there was an increased share of cyclists using the cycle lane after it was coloured. There was a significant increase in cyclists using the cycle lane and a decrease in cyclists using the pavement, which is indicative of how comfortable cyclists feel in the road and also has implications for cyclist-pedestrian interactions.

In addition, we add to the field by considering whether colouring cycle lanes can be defined as nudging and how the concept of nudge can contribute to understanding the mechanisms behind the observed effects. As such the study attempts to place the typically empirically oriented field of transport research within a theoretical framework.

While counting the numbers of cars that stopped in the cycle lane is quite simple, measuring exact distance is not. Efforts were made to ensure as identical measurements as possible before and after, but we cannot rule out that small differences occurred. Nevertheless, we estimate such differences to be smaller than the observed changes.

One strength of this study is the use of survey data in addition to video observations and app data. This made it possible to substantiate some of the behavioural changes with people’s experiences. However, several of the items were quite skewed, indicating a ceiling effect. Thus it might be that potential changes in the positive direction could not be detected, simply because there was little potential to improve the opinions of cyclists, which were positive to start with. Another challenge is that we were not able to conduct surveys in the first phase of the project. As noted, the changes in terms of increased cycling (measured with app data) were most prominent in the first phase, and it might have been that people’s perceived changes of the infrastructure also would have been more prominent then, than what we observed in the latter phase.

The increased use of smart phones gives new opportunities to evaluate infrastructure measures for increased bicycle. As yet there are few other studies using this method (Pritchard et al., 2019). Based on our experiences we can share some limitations of using this approach. First, app data are as vulnerable as traditional surveys for any sample bias, since it is necessary to recruit users to download and activate them. Although passive apps should theoretically be less vulnerable to skewed samples than active apps (where the user has to interact with the app), we saw substantial attrition from recruitment survey to actual app usage. Second, there is the uncertainty resulting from geolocation of trips. All steps involved in the geolocation process involve possible sources of error. These sources of error are random and will not be of great importance when the sample size is large enough. Based on these considerations, we find that aggregated results are less sensitive to sources of error than individual road sections.

With our app data we observe an increase in cycling in the trial streets compared to control streets. We interpret this as a change in route choice among cyclists. As mentioned, we do not believe mode share changes play a large role. The other alternative explanation is that cyclists have not changed their routes, but that we have sampled cyclists with different route preferences in the different years. We cannot rule out this explanation, but by collecting data from a wide range of locations around the city, having a relatively large sample size for a multi-week study, and by using control streets to level out seasonal effects, we have minimised the negative contribution from this limitation.

## Conclusion

This article provides a comprehensive look at the impact of red cycle lanes in Oslo, Norway, and supports previous findings that coloured cycles lanes can impact both motorists’ and cyclists’ behaviour, as well as cyclists’ perceptions of the cycle lane. While we have not looked at accident or injury risks, differences in motorist and cyclist behaviour might impact actual, as well as perceived, safety. These results indicate that using red asphalt affects behaviours and perceptions that are central to the attainment of policy goals for increased cycling. We also argue that coloured cycle lanes can be defined as nudges and that increased salience of cyclists and cycle lanes can explain the observed changes in behaviour and perception.

## Data Availability Statement

The raw data supporting the conclusions of this article will be made available by the authors, without undue reservation.

## Ethics Statement

The studies involving human participants were reviewed and approved by NSD - Norwegian Centre for Research Data. Written informed consent for participation was not required for this study in accordance with the national legislation and the institutional requirements.

## Author Contributions

AF, KK, and HS contributed to the study conception and design, acquisition of data, and critical revision. AF and KK contributed to the analysis and interpretation of data and drafting of manuscript. All authors contributed to the article and approved the submitted version.

## Conflict of Interest

The authors declare that the research was conducted in the absence of any commercial or financial relationships that could be construed as a potential conflict of interest.
